# Publisher Correction: Synergistic electroreduction of carbon dioxide to carbon monoxide on bimetallic layered conjugated metal-organic frameworks

**DOI:** 10.1038/s41467-020-15668-0

**Published:** 2020-04-01

**Authors:** Haixia Zhong, Mahdi Ghorbani-Asl, Khoa Hoang Ly, Jichao Zhang, Jin Ge, Mingchao Wang, Zhongquan Liao, Denys Makarov, Ehrenfried Zschech, Eike Brunner, Inez M. Weidinger, Jian Zhang, Arkady V. Krasheninnikov, Stefan Kaskel, Renhao Dong, Xinliang Feng

**Affiliations:** 1 Center for Advancing Electronics Dresden (Cfaed) and Faculty of Chemistry and Food Chemistry, Technische Universität Dresden, 01062 Dresden, Germany; 2 Helmholtz-Zentrum Dresden-Rossendorf e.V., Institute of Ion Beam Physics and Materials Research, 01328 Dresden, Germany; 3 Shanghai Synchrotron Radiation Facility, Zhangjiang Laboratory, Shanghai Advanced Research Institute, Chinese Academy of Sciences, 201204 Shanghai, China; 4 Fraunhofer Institute for Ceramic Technologies and Systems (IKTS), Maria-Reiche-Strasse 2, 01109 Dresden, Germany; 5 Department of Applied Chemistry, School of Applied and Natural Sciences, Northwestern Polytechnical University, 710129 Xi’an, China; 6 Department of Applied Physics, Aalto University, P.O. Box 11100, FI-00076 Aalto, Finland

**Keywords:** Electrocatalysis, Metal-organic frameworks, Electrocatalysis

Correction to: *Nature Communications* 10.1038/s41467-020-15141-y, published online 16 March 2020.

The original version of this Article did not acknowledge Renhao Dong as a corresponding author. This has now been corrected in both the PDF and HTML versions of the Article.

