# Supplementary material for: Synergistic electroreduction of carbon dioxide to carbon monoxide on bimetallic layered conjugated metal-organic frameworks
Source: Nat Commun. 2020 Mar 16;11:1409. doi: 10.1038/s41467-020-15141-y (PMC7075876; doi:10.1038/s41467-020-15141-y)
Supplement: Supplementary file 1 — Supplementary Information [file 41467_2020_15141_MOESM1_ESM.pdf]

Supplementary Information for

Synergistic Electroreduction of Carbon Dioxide to Carbon Monoxide on Bimetallic Layered Conjugated Metal-Organic Frameworks

Zhong et al.

Supplementary Methods

Computational Details

The process for the CO_2 reduction reaction (CO_2RR) can be written via the following steps[1]:

I) $* + \mathrm{CO_2} + \mathrm{H^+(aq)} + \mathrm{e^-} \longrightarrow * -\mathrm{COOH}$ Equation S1

II) $* -\mathrm{COOH} + \mathrm{H^+}\ (\mathrm{aq}) + \mathrm{e^-} \longrightarrow * -\mathrm{CO} + \mathrm{H_2O}$ Equation S2

III) $* -\mathrm{CO} \longrightarrow * + \mathrm{CO}\ (\mathrm{g})$ Equation S3

where * refers to the catalyst site. Electroreduction of CO_2 may result in a wide range of products. The formation of formate can be excluded in current electrocatalyst system due to the large activation barriers of formate intermediate (Supplementary Fig.2) compared to that for carboxyl intermediate. Generally, formate is formed with high selectivity only at a high overpotential on most materials [1].

The overpotential of CO_2 reduction is defined as:

$$\eta^{CO2RR} = \frac{Max\ [\Delta G_I, \Delta G_{II}, \Delta G_{III}]}{e}$$ Equation S4

where ΔG_i represent the variations of the free energy at i*th* reaction step.

The computational modeling of the reactants, intermediates and products and reaction process involved in the reactions on 2D MOFs was performed by using density functional theory (DFT) within the PBE[2] exchange-correlation functional, as implemented in the VASP[3]. The total energies were converged within 10^{-6} eV/cell. The cut-off energy for plane wave basis was set 500 eV. The Brillouin zone of the supercells was sampled using 4 × 4×1 Monkhorst–Pack grid of *k*-points. All calculations have been performed using spin-polarized setup. London dispersion interactions were added to the total bonding energy as proposed by Grimme in the DFT-D2 method[4].

The binding energy of molecules was calculated as:

$$E_{bin} = E_{Molecule/\text{Catalyst}} - E_{\text{Catalyst}} - E_{Molecule}$$ Equation S5

where the first term is the calculated energy of the molecule adsorbed on catalyst, and the second and third terms are the energy of the catalyst and the isolated molecule, respectively.

Gibbs free energy change (ΔG) of each reaction step is evaluated based on computational hydrogen (CHE) electrode model[5]:

$$\Delta G = \Delta E + \Delta E_{ZPE} - T\Delta S + \Delta G_U$$ Equation S6

where (ΔE) is the reaction energy directly computed from the DFT calculations. The zero-point energy corrections (ΔZPE) and the entropy changes ($T\Delta S$) are obtained from vibrational frequency calculations and standard tables for gas-phase molecules. $\Delta G_U = -neU$ represents the effect of the external potential for the electrochemical step, where n is number of transferred electrons and U is the electrode potential relative to the standard hydrogen electrode. The entropies and ZPE values of the gas-phase molecules are taken from the previous theoretical study on the similar metal surfaces [6]. The CO_2 calculated energy was corrected by 0.45 eV together with a *COOH water correction of 0.25 eV and a *CO water correction of 0.1 eV[7].

Experimental Section

1,2-dicyano-4,5-dimethoxybenzene: 1,2-dibromo-4, 5-dimethoxybenzene (8,87 g, 0.03 mol) and 8.06 g (0.09 mol) of CuCN was refluxed in DMF (110 mL) at 165 ºC for 5 h. After cooling at room temperature, 300 mL of concentrated ammonium hydroxide was added into the above reaction mixture and it was stirred at room temperature for 12 h under air. Then the green solid was obtained by filtration and washing with copious amount of diluted ammonium hydroxide and water. The dry, crude olive-green

product was placed in the thimble of a Soxhlet extractor and extracted with DMF, water and acetone for 3 days. Then the colorless small needles are obtained from acetone.

PcCu-(OMe)$_8$: 1, 2-dibromo-4, 5-dimethoxybenzene (8,87 g, 0.03 mol) and 8.06 g (0.09 mol) of CuCN was refluxed in DMF (110 mL) at 165 ^{o}C for 5 h. After cooling at room temperature, 300 mL of concentrated ammonium hydroxide was added into the above reaction mixture and it was stirred at room temperature for 12 h under air. Then the green solid was obtained by filtration and washing with copious amount of diluted ammonium hydroxide and water. The dry, crude olive-green product was placed in the thimble of a Soxhlet extractor and extracted with methanol and acetone for 1 day. Then the green solid was collected.

PcZn-(OMe)$_8$: 1, 2-dicyano-4, 5-dimethoxybenzene (0.2 g, 0.164) and zinc(II) acetate dihydrate (0.058 g, 0.266) was dissolved into dimethylaminoethanol (DMAE, 5 mL) under Ar and heating at 110 ^{o}C for 24 h. After cooling to room temperature, a mixture of methanol and water (20 mL, 3/1in vol) was added the above solution. Then, PcZn-(OMe)$_8$ was obtained by filtration and washing with methanol and acetone, as well as vacuum drying.

PcCu-(OH)$_8$: PcCuOMe (2 g) was dissolved into 300 mL of dry dichloromethane, and boron tribromide (4.8 mL) was slowly added under Ar atmosphere. After stirring at room temperature for 2 h, the mixture was refluxed for 20 h (50 ^{o}C). After cooling down, 50 mL of methanol was slowly added into the mixture. Then the precipitate was filtered and washed with MeOH, water, and dichloromethane, centrifuged many times with water, MeOH, and dichloromethane until the supernatant became almost transparent, as well as dried under vacuum.

PcZn-(OH)$_8$: PcZnOMe (160 mg) was dissolved into 10 mL of dry dichloromethane, and boron

tribromide (0.76 mL) was added under Ar atmosphere. The mixture was stirring at room temperature for 3 days. Afterward, 10 mL of methanol was slowly added into the mixture. Then the precipitate was filtered and washed with MeOH, water, and dichloromethane, centrifuged many times with water, MeOH, and dichloromethane until the supernatant became almost transparent, as well as dried under vacuum.

Reagents: All solvents, reagents and chemicals were purchased from commercial suppliers (Sigma-Aldrich, TCI and abcr GmbH.) and used without further purification unless specially addressed. All the reactions were performed using the standard vacuum-line and Schleck techniques under argon or vacuum.

Supplementary Tables

Supplementary Table 1 | Calculated lattice parameters of different PcM-O_8-M1 unit cells.

MOF	PcCu-O_8-Zn	PcZn-O_8-Zn	PcCu-O_8-Cu	PcZn-O_8-Cu
Lattice parameter (Å)	18.41	18.51	18.20	18.26

Supplementary Table 2 | Binding energies (eV) of *COOH on M site of PcM-O_8-M1.

MOF	PcCu-O_8-Cu	PcCu-O_8-Zn	PcZn-O_8-Cu	PcZn-O_8-Zn
COOH*	-0.94	-1.18	-1.15	-1.19

Supplementary Table 3 | Binding energies (eV) of *COOH on M1 site of PcM-O_8-M1.

MOF	PcCu-O_8-Cu	PcCu-O_8-Zn	PcZn-O_8-Cu	PcZn-O_8-Zn
COOH*	-1.40	-1.62	-1.44	-1.43

Supplementary Table 4 | Binding energies (eV) of hydrogen atom on M site of PcM-O_8-M1.

MOF	PcCu-O_8-Cu	PcCu-O_8-Zn	PcZn-O_8-Cu	PcZn-O_8-Zn
*H	-2.15	-2.42	-2.27	-2.26

Supplementary Table 5 | Binding energies (eV) of hydrogen atom on M1 site of PcM-O_8-M1.

MOF	PcCu-O_8-Cu	PcCu-O_8-Zn	PcZn-O_8-Cu	PcZn-O_8-Zn
*H	-0.79	-1.09	-0.81	-0.92

Supplementary Table 6 | Overpotential, η^{CO2RR}(eV) of CO_2RR on M and M1 sites (PcM-O_8-M1).

CO_2RR	PcCu-O_8-Cu	PcCu-O_8-Zn	PcZn-O_8-Cu	PcZn-O_8-Zn
On M site	2.16	1.85	1.87	1.83
On M1 site	1.65	1.41	1.66	1.60

Supplementary Table 7 | Overpotential, η^{HER}(eV) of HER on M and M1 sites (PcM-O_8-M1).

HER	PcCu-O_8-Cu	PcCu-O_8-Zn	PcZn-O_8-Cu	PcZn-O_8-Zn
On M site	0.50	0.23	0.38	0.39
On M1 site	1.68	1.38	1.66	1.55

Supplementary Table 8 | Comparison of CO_2RR performance of our 2D MOF with other related materials.

Materials	Electrolyte	j (mA cm^{-2})[a]	E (V vs RHE)[b]	FE for CO (%)	TOF s^{-1}	Reference
PcCu-O_8-Zn /CNT	0.1 M $KHCO_3$	8	-0.7	88	0.39	This work
$Al_2(OH)_2$TCPP-Co	0.5 M K_2CO_3	2.3	-0.7	76	0.05	J. Am. Chem. Soc. 2015, 137, 14129-14135.
Fe_MOF-525	1 M $TBAPF_6$ MeCN	5.9	-1.3[c]	54	0.13	ACS Catal. 2015, 5, 6302−6309
COF-300-AR on Ag foil	0.1 M $KHCO_3$	2.5	-0.85	80	/	Chem, 2018, 4, 1–14.
Fe/NG-750	0.1 M $KHCO_3$	2.75	-0.6	80	/	Adv. Energy Mater. 2018, 1703487
ZIF-A-LD/CB	0.1 M $KHCO_3$	3.2	-1	75	/	Angew.Chem.Int.Ed.2019,58,4041 – 4045
PD-Zn/Ag	0.1 M $KHCO_3$	-9.7	-1.2	74	/	Angew.Chem.Int.Ed.2019,58,2256 –2260
h-Zn	0.5 M $KHCO_3$	-9.5	-0.95	85.4	/	Angew.Chem.Int.Ed.2016,55,9297 –9300
Ag@Al-PMOF	0.1 M $KHCO_3$	-0.6	-1.1	55.8	*0.014*	Angew.Chem.Int.Ed.2019,58,12632 -12639
COF-366-Co	0.5 M $KHCO_3$	-5 mA mg^{-1}	-0.67	90	0.69	Science 2015 349, 1208-1213.
COF-367-Co(10%)	0.5 M $KHCO_3$	-4.5 mA mg^{-1}	-0.67	70	2.6	

$TOF = v_{CO} \quad * flow\ rate * \frac{P_0}{RTn_{cat}}$ Here, n_{cat} is the amount of the Zn of PcCu-O_8-Zn.

[a] Current density is the highest partial current density for CO over the applied potentials.

[b] The potential is which the catalyst shows the highest FE. [c] V vs. NHE

Supplementary Figures

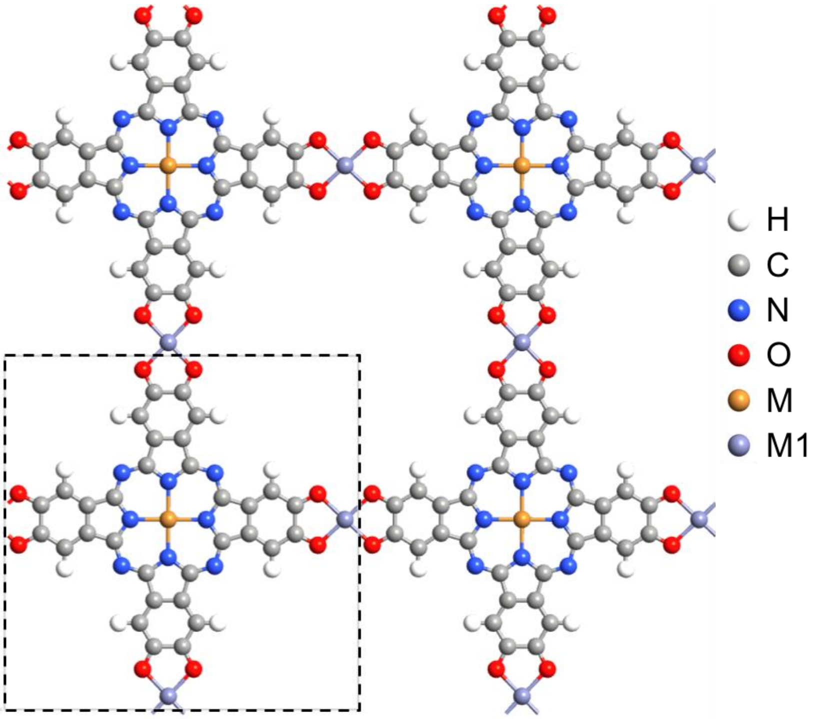

Supplementary Figure 1 | Model structure of 2D *c*-MOFs. The atomistic structure of PcM-O_8-M1 and the dashed rectangular indicates the unit cell.

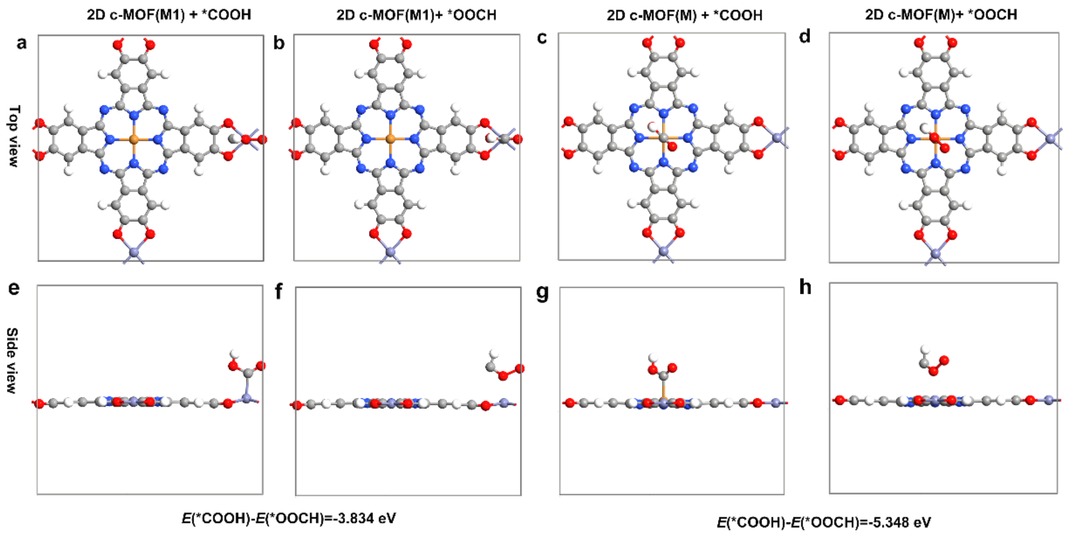

Supplementary Figure 2 | Model structures of 2D *c*-MOFs with the intermediates (*COOH and *OOCH on M1 and M sites. The top view atomistic structure of the interaction of PcM-O_8-M1 carboxyl intermediate (*COOH, **a**: M1, **c**: M) and formate intermediate (*OOCH **b**: M1, **d**: M); The side view atomistic structure of the interaction of PcM-O_8-M1 carboxyl intermediate (*COOH, **e**: M1, **g**: M) and formate intermediate (*OOCH, **f**: M1, **h**: M).

Generally, formate is formed with high selectivity only at a high overpotential on most materials. Supplementary Fig. 2 shows that the calculated barrier energy for the formation of the formate (*OOCH) intermediate is much higher than for the formation of the carboxyl intermediate (*COOH) on PcM-O_8-M1 catalyst with an energy difference of 3.834 eV on M1 site and 5.348 eV on M site. Therefore, the formation of formate was not favorable in PcM-O_8-M1 system.

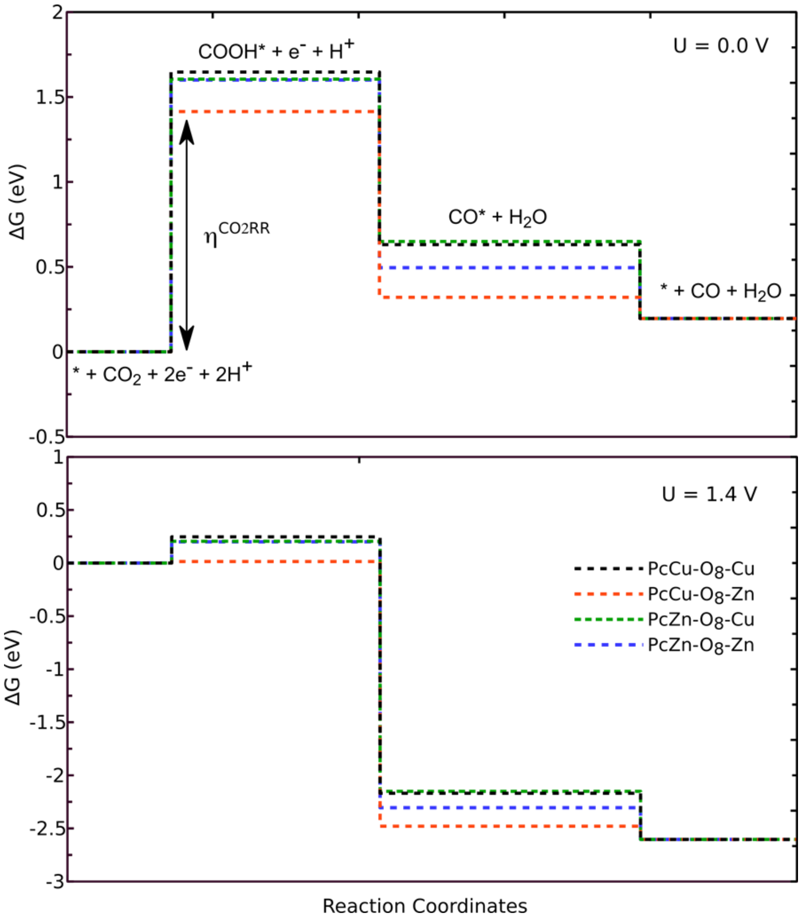

Supplementary Figure 3 | CO_2RR process analysis. Free energy profiles of CO_2RR on M1 site (PcM-O_8-M1). The results are obtained at U = 0.0 V and U = 1.40 V.

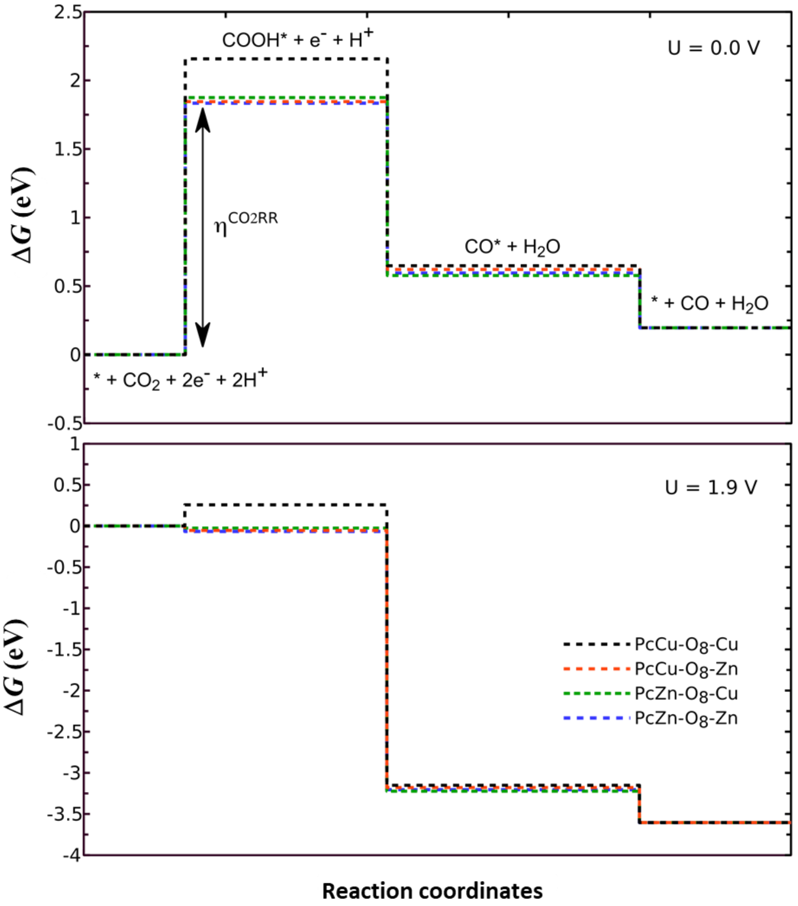

Supplementary Figure 4 | CO_2RR process analysis. Free energy profiles of CO_2RR on M site (PcM-O_8-M1). The results are obtained at U = 0.0 V and U = 1.90 V.

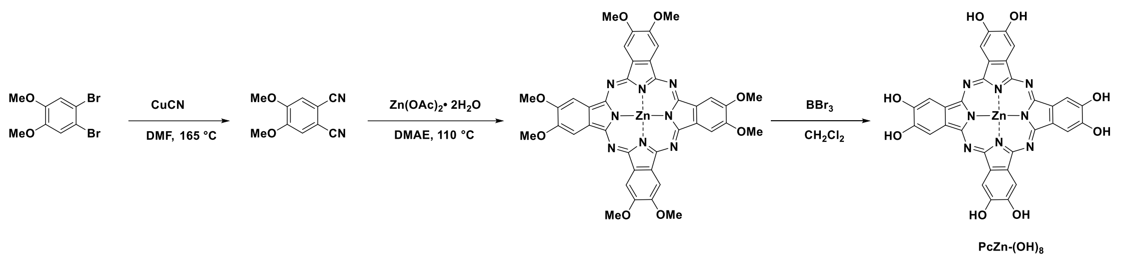

Supplementary Figure 5| Ligand synthesis. The typical synthesis process of PcZn-OH_8.

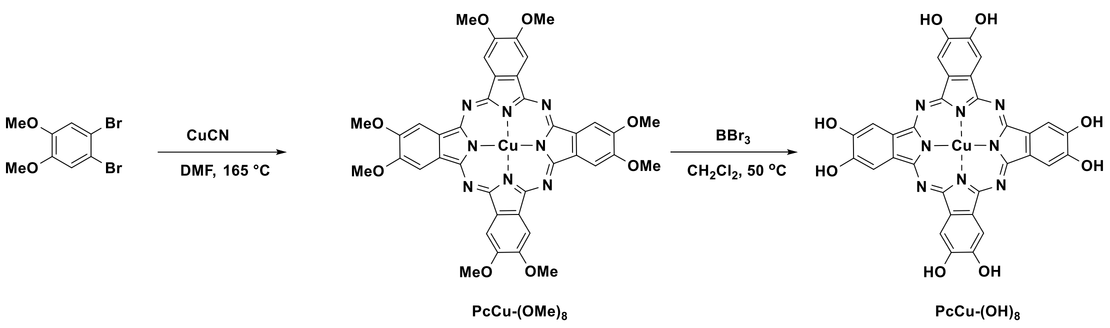

Supplementary Figure 6 | Ligand synthesis. The typical synthesis process of PcCu-OH_8.

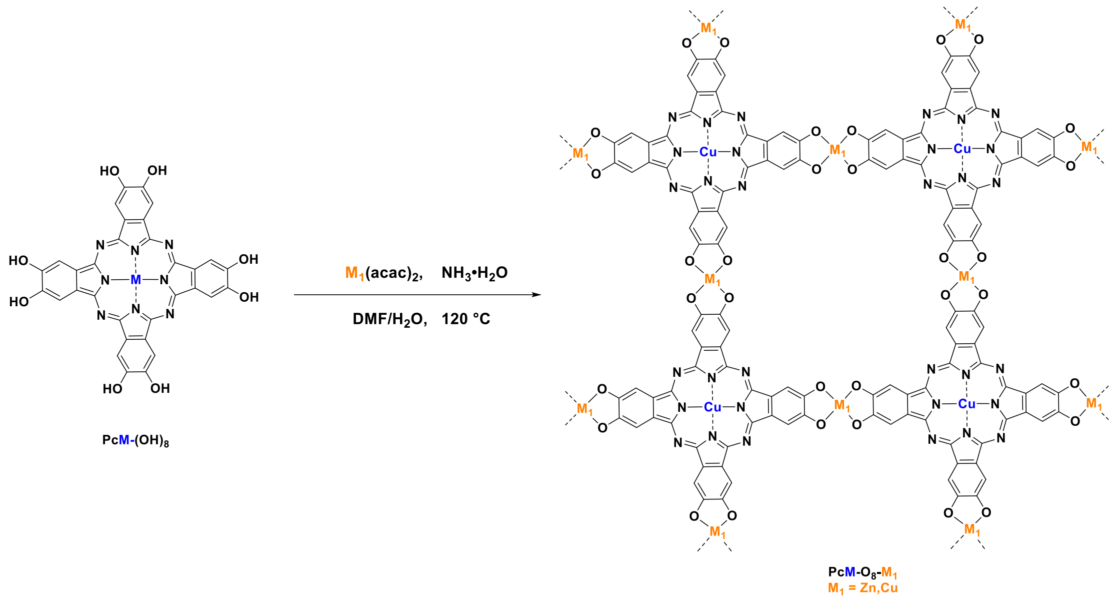

Supplementary Figure 7 | 2D *c*-MOFs synthesis. The typical synthesis process of PcM-O_8-M1 (M/M1=Cu and Zn).

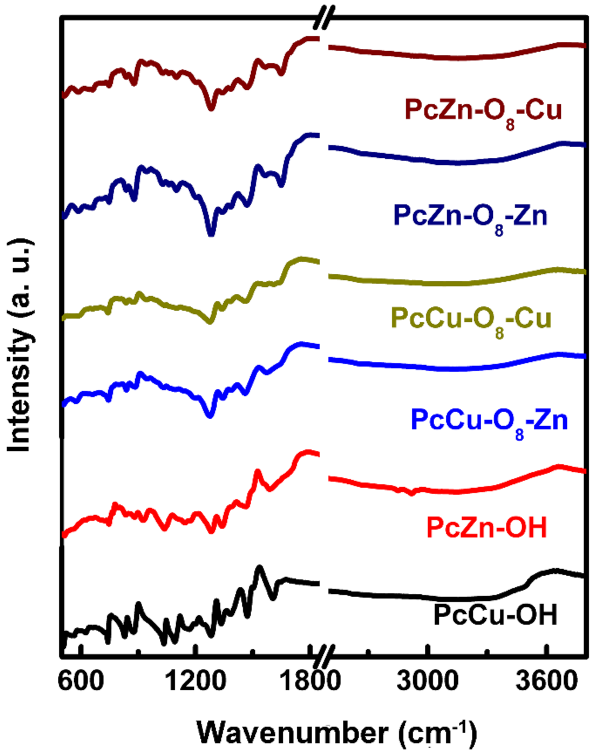

Supplementary Figure 8 | Structure characterization. FT-IR spectra of PcCu-$(OH)_8$, PcZn-$(OH)_8$ and PcM-O_8-M1 (M/M1=Cu, and Zn).

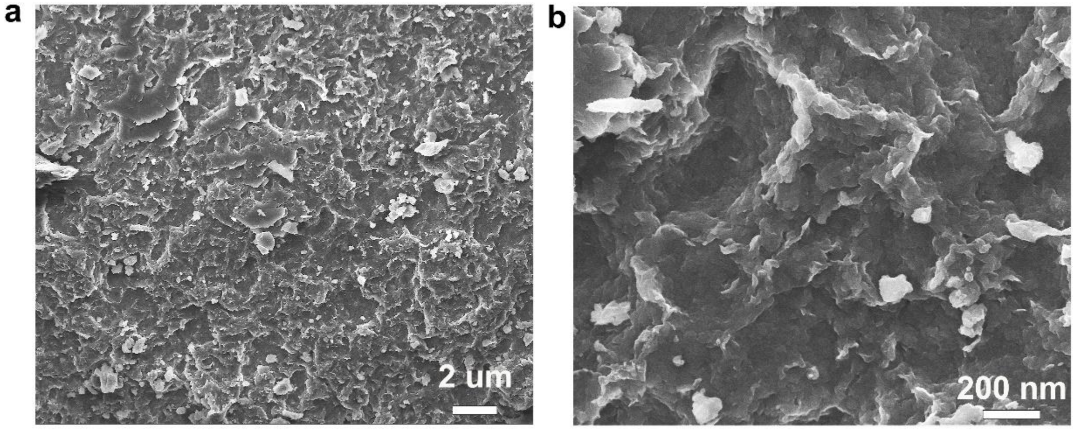

Supplementary Figure 9 | Morphology of PcCu-O_8-Zn. a) Low amplification SEM image. **b)** High amplification SEM image.

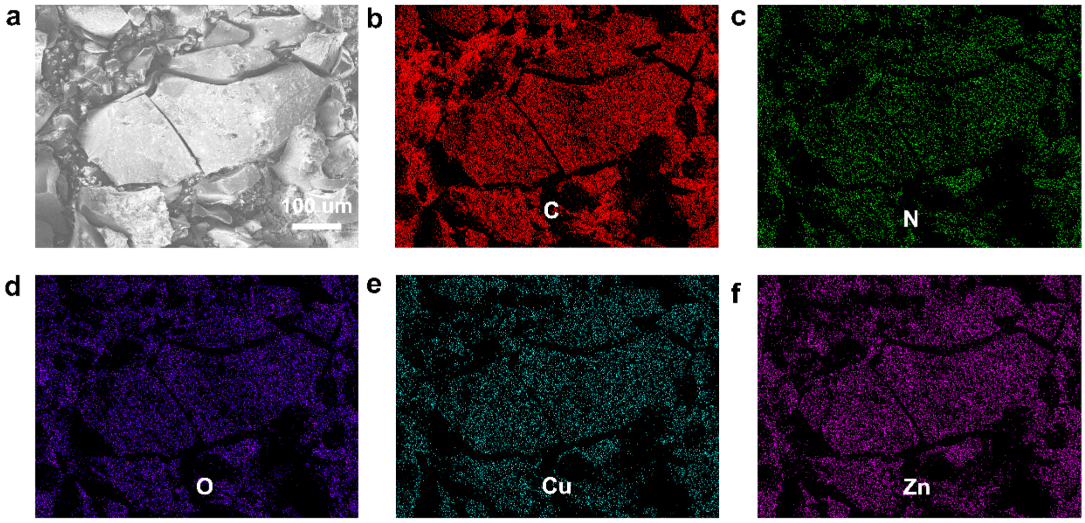

Supplementary Figure 10 | Morphology and elemental analysis. a) SEM image of PcCu-O_8-Zn. **b-f)** Corresponding element mapping images of C, N, O, Cu and Zn.

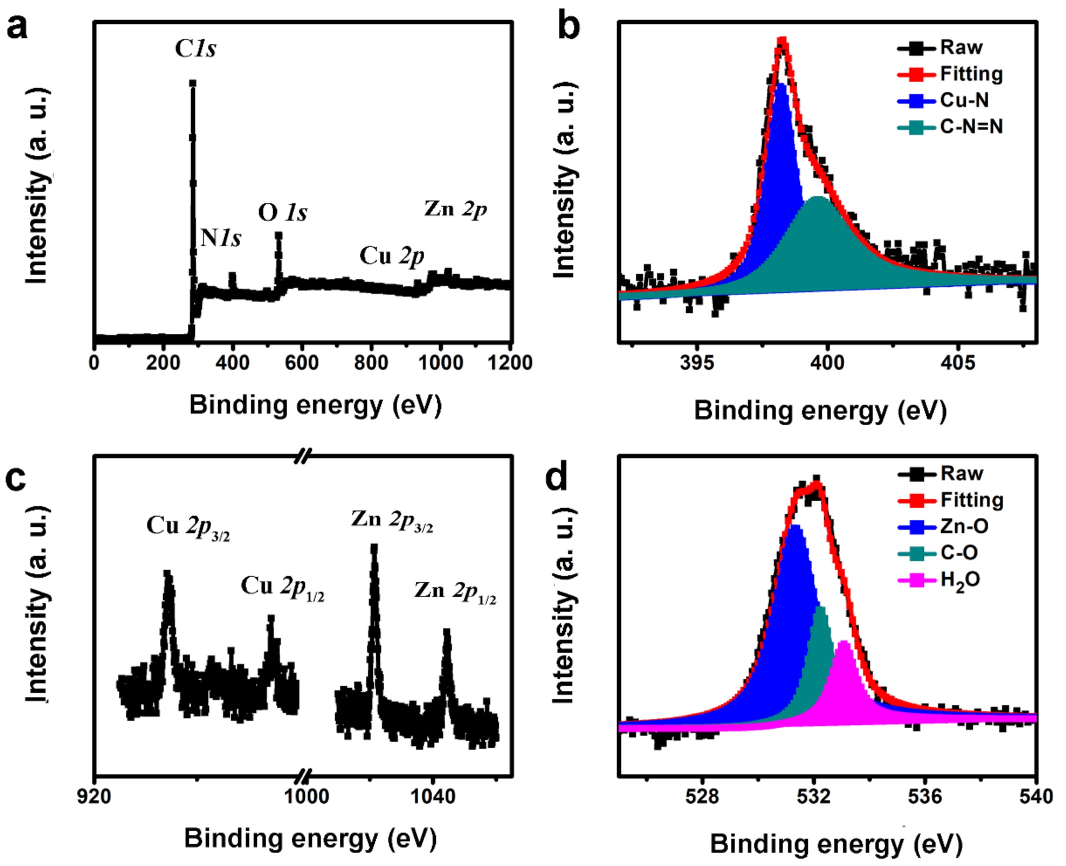

Supplementary Figure 11 | Chemical structure analysis. Survey (**a**), N*1s* (**b**), Cu *2p*/Zn *2p* (**c**) and O *1s* (**d**) XPS spectra of PcCu-O_8-Zn.

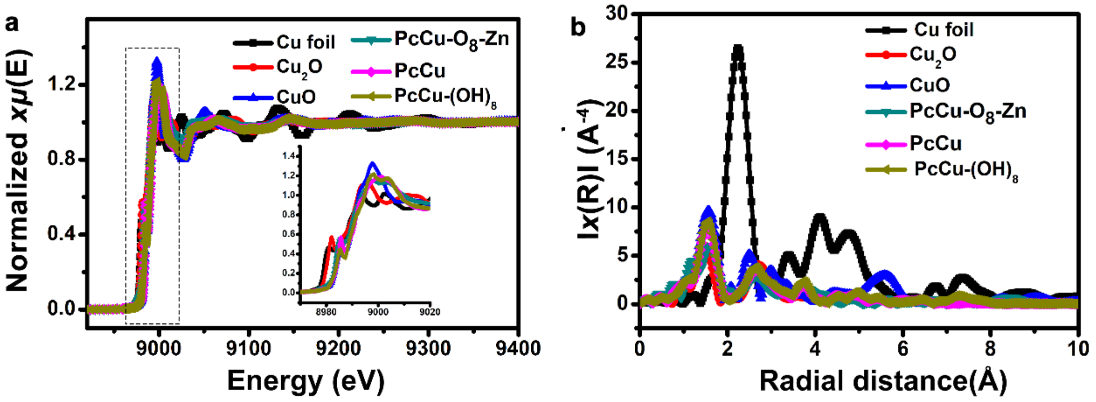

Supplementary Figure 12 | Chemical environmental measurement. XANES of Cu K-edge **(a)** and Fourier transform EXAFS spectra (**b)** of Cu foil, Cu_2O, CuO, PcCu, PcCu-$(OH)_8$ and PcCu-O_8-Zn.

Fourier transform EXAFS spectra show the partial radial distribution functions for Cu atom of PcCu-O_8-Zn, and clearly demonstrate the characteristic Cu-N coordination in PcCu-O_8-Zn by an intensive peak at around 1.56 Å, which is similar with that of PcCu and PcCu-$(OH)_8$. The absence of obvious structural peaks at 2.23 Å reveals that no heavy backscattering atoms (Cu) are bounding to Cu sites in PcCu-O_8-Zn.

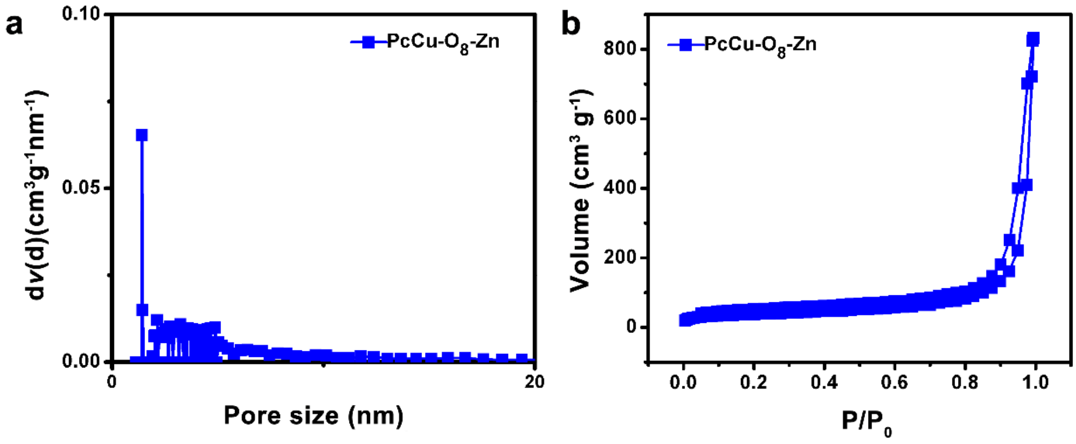

Supplementary Figure 13 | Pore properties. a) Pore size distribution of PcCu-O_8-Zn. **b)** Nitrogen absorption-desorption isotherm of PcCu-O_8-Zn.

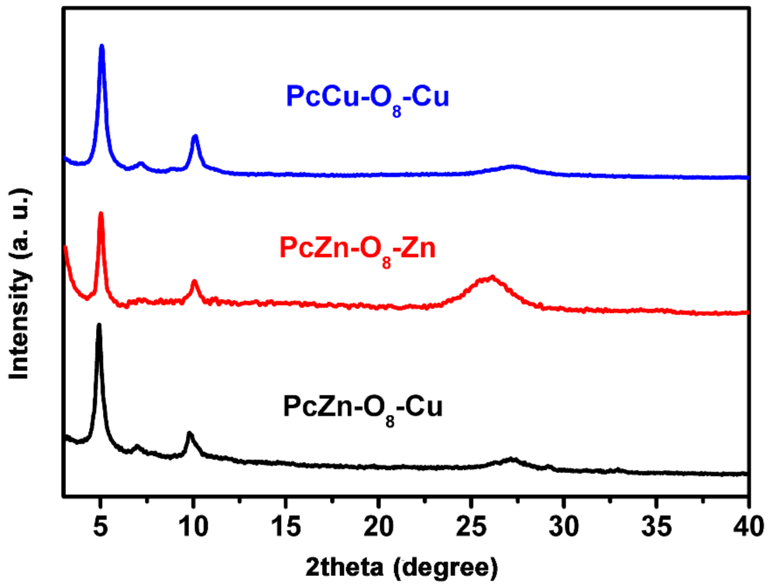

Supplementary Figure 14 | Structure analysis. XRD patterns of PcM-O_8-M1 (M/M1=Cu, and Zn).

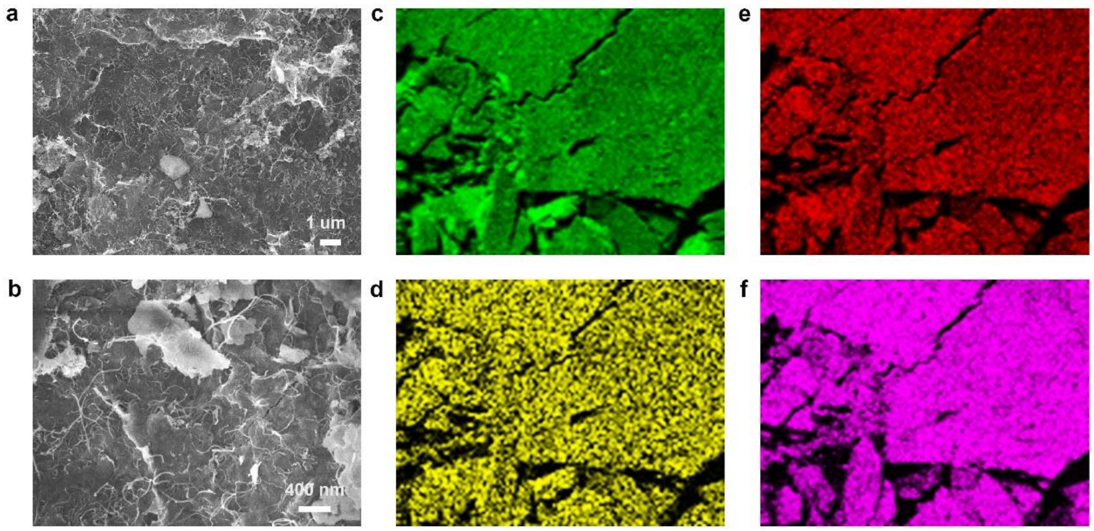

Supplementary Figure 15 | Elemental analysis. a, b) SEM image of PcZn-O_8-Zn/CNT. **c-f)** Corresponding element mapping images of C (**c**), N (**d**), O (**e**) and Zn (**f**).

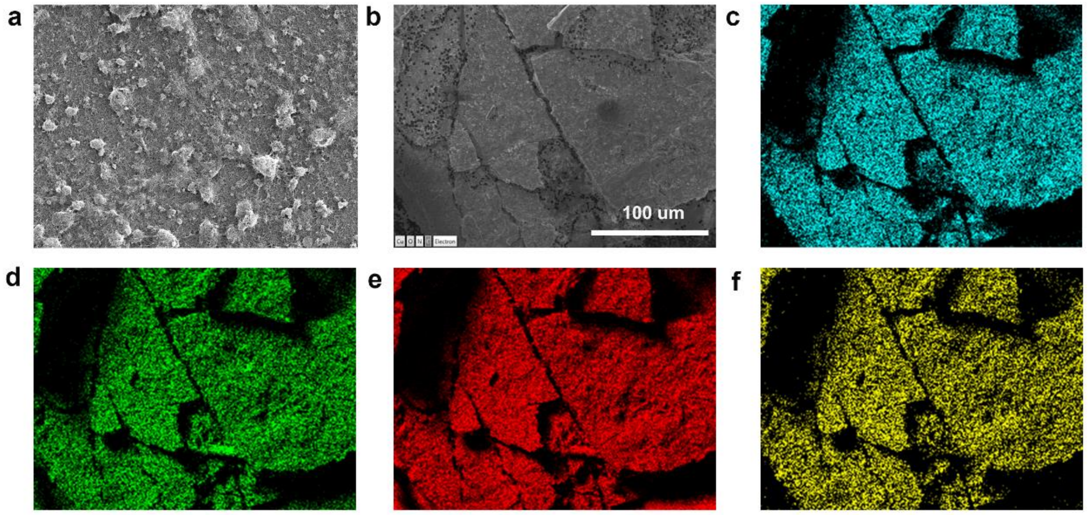

Supplementary Figure 16 | Elemental analysis. a, b) SEM image of PcCu-O_8-Cu/CNT. **c-f)** Corresponding element mapping images of C (**c**), N (**d**,) O (**e**) and Cu (**f**) elements.

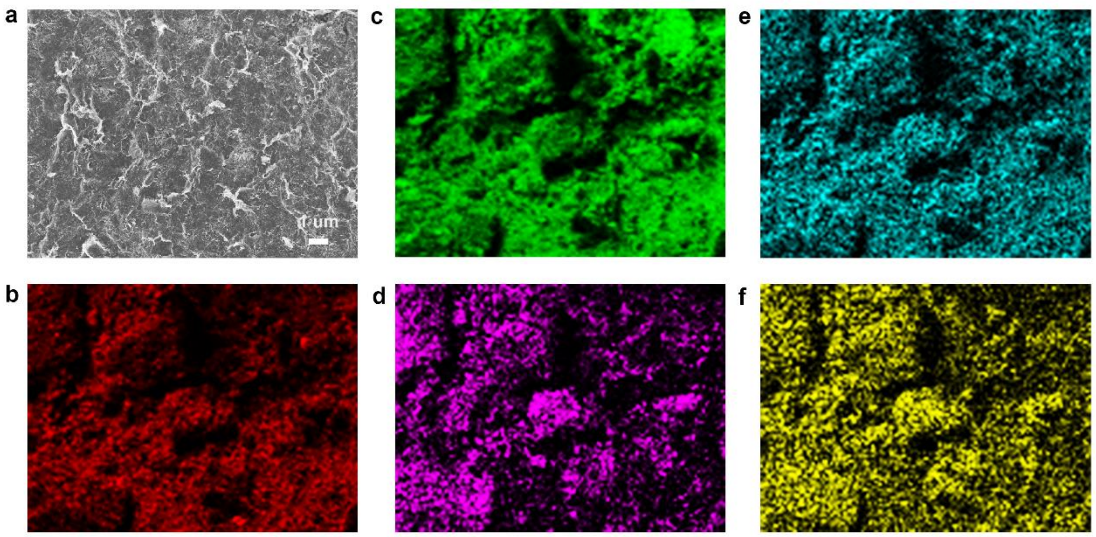

Supplementary Figure 17 | Elemental analysis. a) SEM image of PcZn-O_8-Cu/CNT. **b-f)** Corresponding element mapping images of O (**b**), C (**c**), Zn (**d**), N (**e**,) and Cu (**f**) elements.

Supplementary Fig. 14 shows intensive peaks at 5.0°, 7.1° and 10.1 ° for PcZn-O_8-Zn, PcZn-O_8-Cu and PcCu-O_8-Cu, assigned to (100), (110) and (200) plane, respectively, confirming their excellent crystallinity. SEM images (Supplementary Fig. 15a, 16a and 17a) display their aggregated particle morphology mixing with CNTs. And the element mapping measurement (Supplementary Fig 15c-f, 16 c-f and 17b-f) confirm the well dispersion of all elements over the whole structure (C, N, O, Zn for PcZn-O_8-Zn/CNT; C, N, O, Cu for PcCu-O_8-Cu/CNT; C, N, O, Zn, Cu for PcZn-O_8-Cu/CNT). It is thus clear that the PcZn-O_8-Zn/CNT, PcZn-O_8-Cu/CNT and PcCu-O_8-Cu/CNT were successfully prepared.

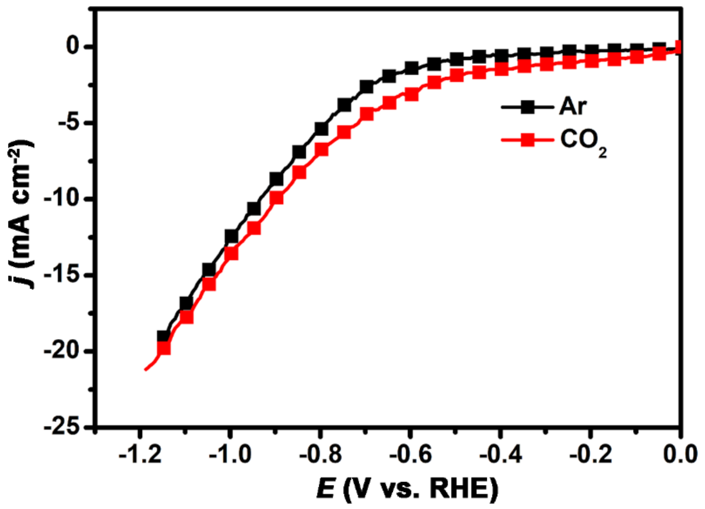

Supplementary Figure 18 | CO_2RR performance. CV curves of PcCu-O_8-Zn/CNT in Ar- and CO_2-saturated 0.1 M $KHCO_3$.

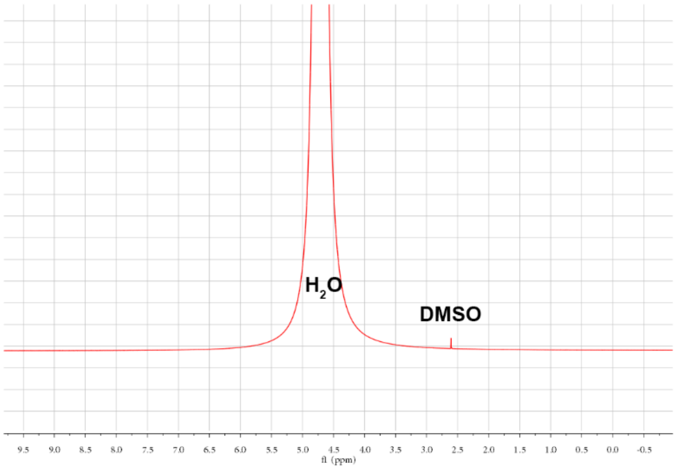

Supplementary Figure 19 | Product analysis. NMR spectrum of the electrolyte for PcCu-O_8-Zn/CNT after 12 h constant potential electrolysis at -0.7 V vs. RHE.

In the NMR spectra, no signal of liquid products (e. g. HCOOH) is observed for PcCu-O_8-Zn/CNT, indicating no liquids are generated at PcCu-O_8-Zn/CNT electrocatalyst under 12 h constant potential electrolysis at -0.7 V vs. RHE.

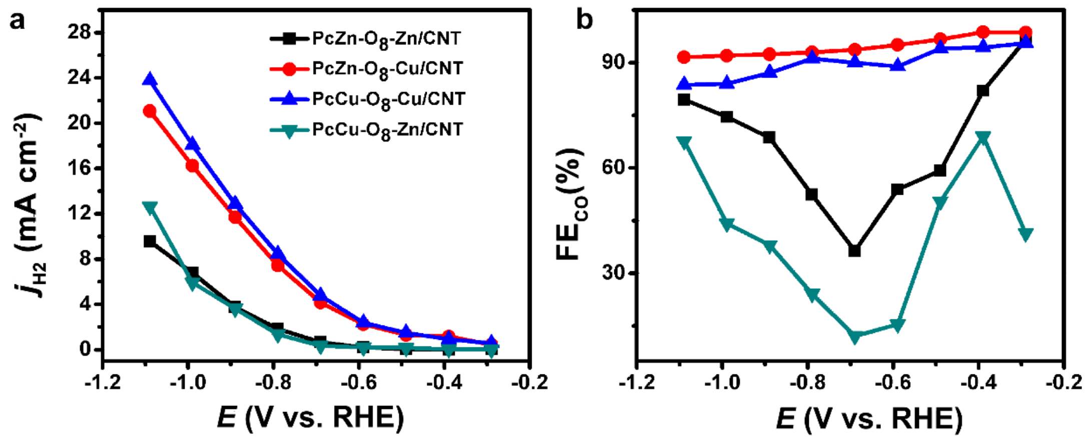

Supplementary Figure 20 | CO_2RR performance. a) Partial current of H_2 for PcCu-O_8-Zn/CNT, PcCu-O_8-Cu/CNT, PcZn-O_8-Zn/CNT and PcZn-O_8-Cu/CNT at different potentials in CO_2-saturated 0.1 M $KHCO_3$. **b)** Faradaic efficiency of H_2 for PcCu-O_8-Zn/CNT, PcCu-O_8-Cu/CNT, PcZn-O_8-Zn/CNT and PcZn-O_8-Cu/CNT at different potentials.

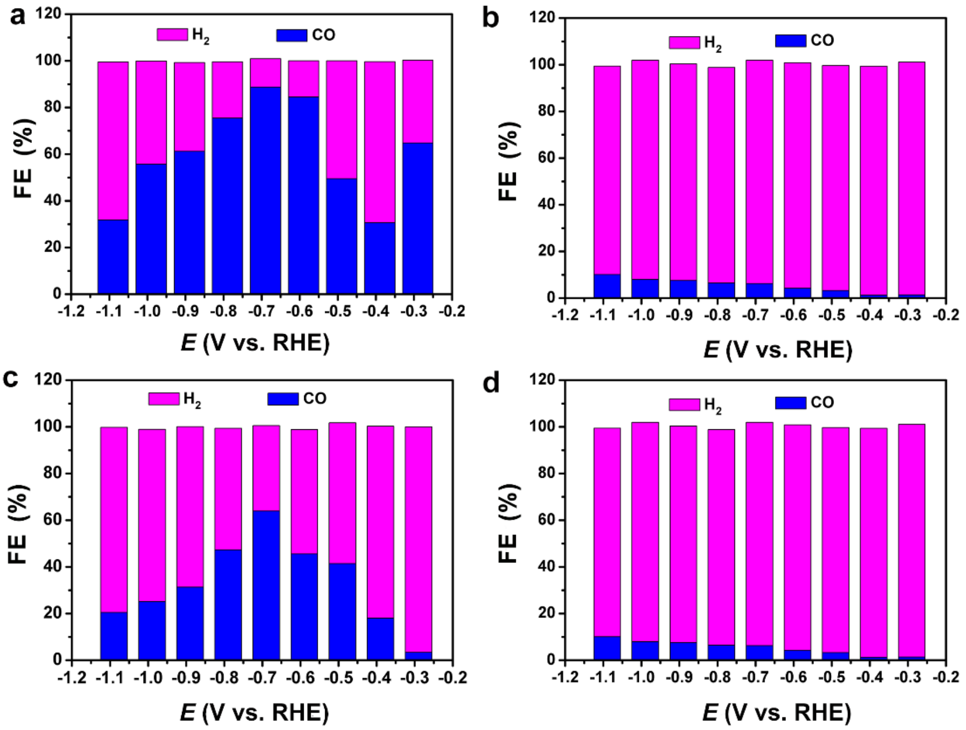

Supplementary Figure 21 | CO_2RR performance. Faradaic efficiency of H_2 and CO for PcCu-O_8-Zn/CNT (**a**), PcCu-O_8-Cu/CNT (**b**), PcZn-O_8-Zn/CNT (**c**) and PcZn-O_8-Cu/CNT (**d**) at different potentials.

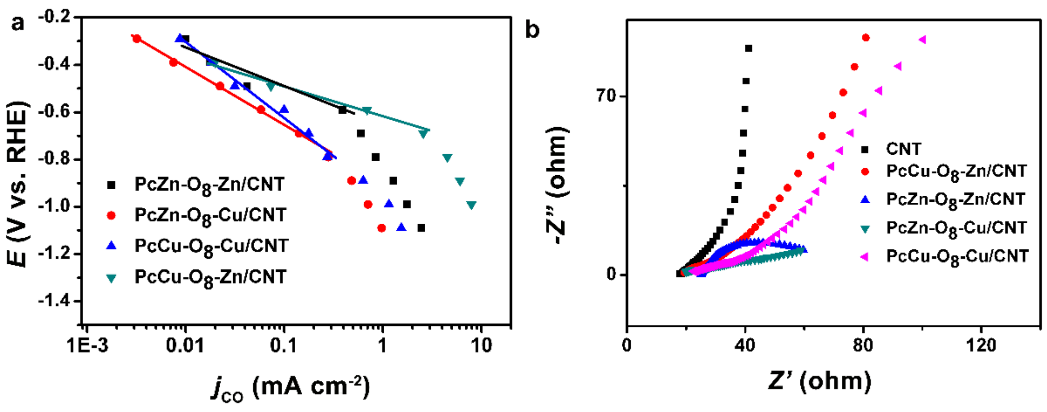

Supplementary Figure 22 | CO_2RR performance. a) Tafel plots of PcCu-O_8-Zn/CNT, PcCu-O_8-Cu/CNT, PcZn-O_8-Zn/CNT and PcZn-O_8-Cu/CNT toward CO_2RR to CO. **b)** EIS of CNT, PcCu-O_8-Zn/CNT, PcCu-O_8-Cu/CNT, PcZn-O_8-Zn/CNT and PcZn-O_8-Cu/CNT in CO_2-saturated 0.1 M $KHCO_3$.

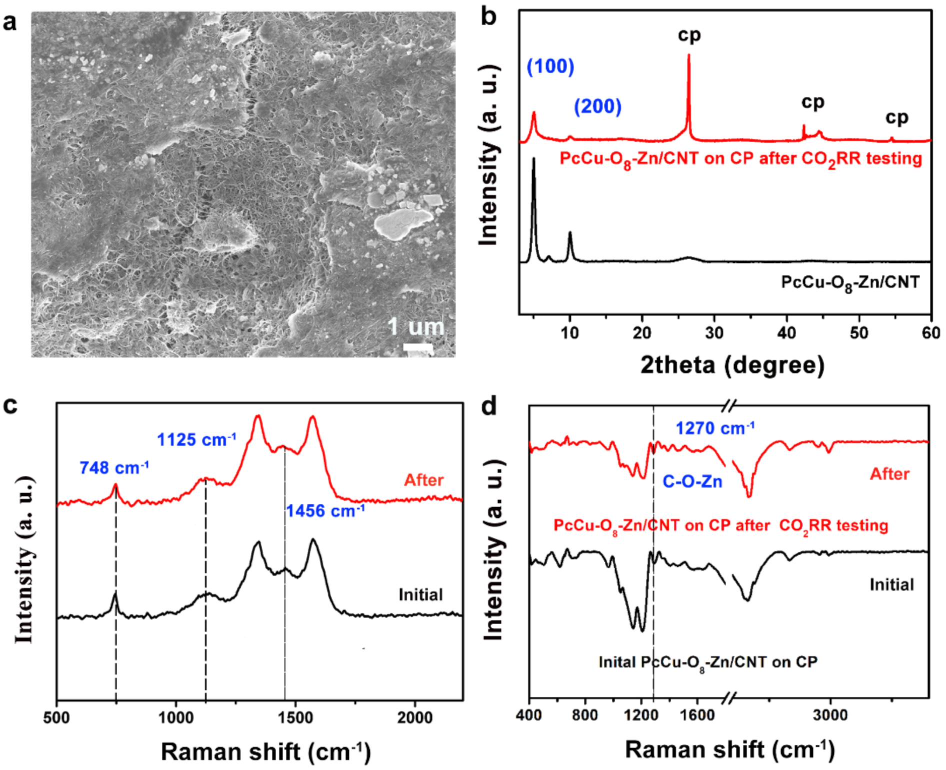

Supplementary Figure 23 | Stability studies of PcCu-O_8-Zn/CNT sample after CO_2RR testing. a) SEM image. **b)** XRD patterns. **c)** Raman spectra. **d)** FT-IR spectra.

As shown in Supplementary Fig. 9 and 23a, SEM image indicate that there is no obvious changes in the morpholoyg of PcCu-O_8-Zn/CNT after the long-term CO_2RR testing. Moreover, the maintained peaks including (100), (200) plane in the XRD patterns of PcCu-O_8-Zn/CNT after the long-term CO_2RR testing (Supplementary Fig. 23b), further confirm the retention of crystallinity. The stability of PcCu-O_8-Zn/CNT after the long-term CO_2RR testing is also gleaned from absence of spectral changes in the Raman spectra before and after applying electrolysis. No obvious shift of the band at 748, 1125 and 1456 cm^{-1} were observed in the Raman spectra in Supplementary Fig. 23c, which belong to macrocycle ring stretch, pyrrole breating and metal associatd C-N-C vibration, respectively. Comparison of the typical peak of C-O-Zn at 1272 cm^{-1} in the FT-IR spectra (Supplementary Fig. 23d) of PcCu-O_8-Zn/CNT before and after the long-term CO_2RR testing further demonstrated the integrity of the coordination networks and the stability of PcCu-O_8-Zn/CNT.

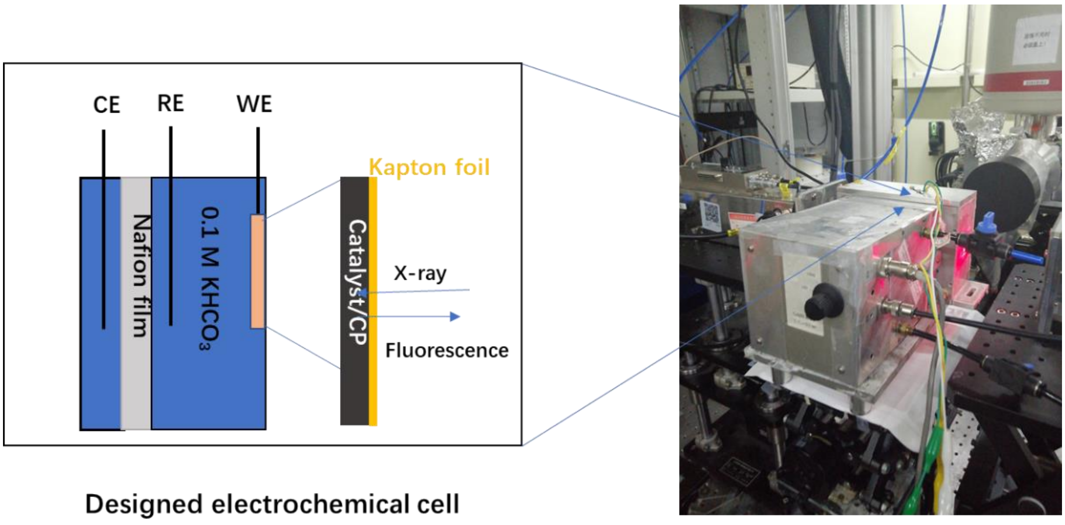

Supplementary Figure 24 | *Operando* XAS setup used in CO_2RR experiments. CE, RE, and WE stand for counter, reference and working electrodes, respectively.

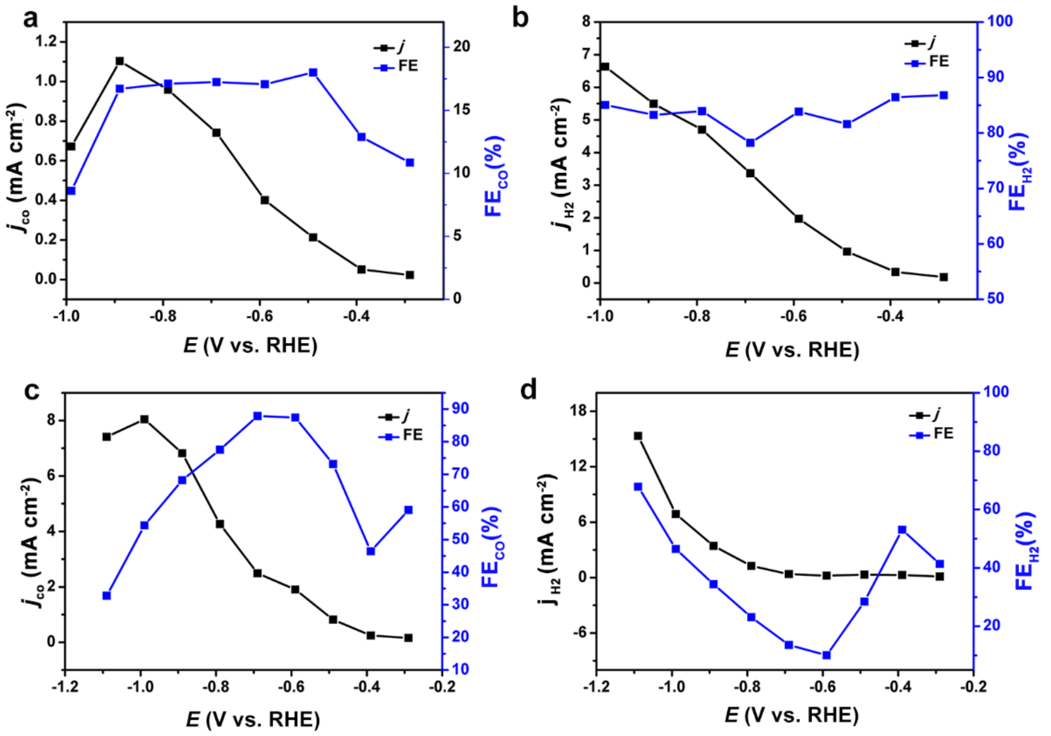

Supplementary Figure 25 | Catalytic activity of Au/CP and PcCu-O$_8$-Zn/CNT on Au/CP. a) j_{CO} and FE $_{CO}$ for Au/CP. **b)** j_{H2} and FE_{H2} for Au/CP. **c)** j_{CO} and FE_{CO} for PcCu-O$_8$-Zn/CNT on Au/CP. **d)** j_{H2} and Fe_{H2} for PcCu-O$_8$-Zn/CNT on Au/CP in CO_2-saturated 0.1 M $KHCO_3$ at different potentials.

To check if the Au substrate affects the CO_2RR performance of 2D *c*-MOF catalysts, we additionally prepared the nano Au on carbon paper (Au/CP) as the current collector and the performance of Au/CP and PcCu-O$_8$-Zn/CNT on Au/CP was evaluated. As shown in Supplementary Fig. 25a-b, Au/CP displays low CO_2RR performance with low Faradaic efficiency (FE) for CO (<16 %) and high FE toward H_2 (close to 90 %) over the whole applied potentials. However, the PcCu-O$_8$-Zn/CNT on Au/CP exhibits much higher CO_2RR performance with Faradaic efficiency (FE) for CO of 89 % at -0.7 V vs. RHE. The partial current density of CO for PcCu-O$_8$-Zn/CNT on Au/CP also presents a maximum value at -1.0 V vs. RHE, while H_2 generation (j_{H2}) displays a steady rise over the increased overpotential because of the competitive reactivity between the CO_2RR and HER as well as the limitation of the transport of CO_2 to the catalytic sites. This trend of current density and FEco for PcCu-O$_8$-Zn/CNT on Au/CP is similar with

PcCu-O_8-Zn/CNT on CP (Supplementary Fig. 25c-d), indicating the major contribution of PcCu-O_8-Zn/CNT to the CO_2RR performance of the whole electrode. Therefore, operando SEIRA analysis performed with 2D *c*-MOFs deposited onto a nanostructured Au surface indeed demonstrates the CO_2RR activity of 2D *c*-MOFs

Supplementary References:

1 Hansen, H. A., Varley, J. B., Peterson, A. A. & Nørskov, J. K. Understanding trends in the electrocatalytic activity of metals and enzymes for CO_2 reduction to CO. *J. Phys. Chem. Lett.* **4**, 388-392 (2013).

2 Perdew, J. P., Burke, K. & Ernzerhof, M. Perdew, Burke, and Ernzerhof Reply. *Phys. Rev. Lett.* **80**, 891-891 (1998).

3 Kresse, G. & Hafner, J. Ab initio molecular dynamics for liquid metals. *Phys. Rev. B* **47**, 558-561 (1993).

4 Grimme, S. Semiempirical GGA-type density functional constructed with a long-range dispersion correction. *J. Comput. Chem.* **27**, 1787-1799 (2006).

5 Nørskov, J. K. *et al.* Origin of the overpotential for oxygen reduction at a fuel-cell cathode. *J. Phys. Chem. B,* **108**, 17886-17892 (2004).

6 Valdés, Á., Qu, Z. W., Kroes, G. J., Rossmeisl, J. & Nørskov, J. K. Oxidation and photo-oxidation of water on TiO_2 Surface. *J. Phys. Chem. C* **112**, 9872-9879 (2008).

7 Peterson, A. A., Abild-Pedersen, F., Studt, F., Rossmeisl, J. & Nørskov, J. K. How copper catalyzes the electroreduction of carbon dioxide into hydrocarbon fuels. *Energy Environ. Sci.* **3**, 1311-1315 (2010).
